# Comparative Assessment of Postoperative Pain After Three Irrigation Techniques in Single-Rooted Teeth With Symptomatic Irreversible Pulpitis and Symptomatic Apical Periodontitis: A Randomized Controlled Trial

**DOI:** 10.7759/cureus.65618

**Published:** 2024-07-29

**Authors:** Pravin Kumar, Sumit Kumar Yadav, Vinay Kumar Chugh, Rajat Sharma, Karishma Pathak, Arunkumar Duraisamy

**Affiliations:** 1 Conservative Dentistry and Endodontics, All India Institute of Medical Sciences, Jodhpur, Jodhpur, IND; 2 Orthodontics and Dentofacial Orthopedics, All India Institute of Medical Sciences, Jodhpur, Jodhpur, IND; 3 Conservative Dentistry and Endodontics, Manav Rachna Dental College, Faridabad, IND

**Keywords:** side vented needle, postoperative pain, negative apical pressure, irrigation, endovac, endoactivator, apical periodontitis

## Abstract

Aim

This study aimed to assess the impact of positive pressure, negative pressure (EndoVac), and sonic-activated irrigation (EndoActivator) on postoperative pain with symptomatic irreversible pulpitis and symptomatic apical periodontitis. The hypothesis tested the superiority of negative pressure irrigation in reducing pain and analgesic requirements.

Methodology

Forty-eight eligible patients meeting inclusion criteria were enrolled, ensuring comprehension through verbal and written patient information sheets. The sample size calculation, based on prior data, determined 14 teeth per group with consideration for potential dropouts, resulting in 16 teeth per group. Inclusion criteria included healthy individuals aged 16-65 years with single-rooted teeth diagnosed with symptomatic irreversible pulpitis and symptomatic apical periodontitis, while exclusion criteria comprised recent analgesic intake, pregnancy, lactation, and specific dental conditions. Participants were allocated to three groups using computer-generated block randomization with allocation concealment via sequentially numbered opaque sealed envelopes. While blinding of the operator was not feasible, patient and assessor blinding was ensured. Preoperative data collection included patient demographics, tooth details, and pain intensity assessed on a Visual Analogue Scale (VAS). Root canal therapy procedures, conducted in two visits, included instrumentation and irrigation using 3% NaOCl across three groups: positive pressure irrigation, negative pressure irrigation, and sonic activation. Postoperative pain and analgesic intake were evaluated using VAS at specific intervals. One assessor tabulated and analyzed all the information.

Results

Postoperative pain assessments revealed that the EV group experienced the lowest pain levels, followed by the EA and SVN groups, with significant differences observed at six and 24 hours postoperatively (p < 0.05). Analgesic requirements correlated with pain levels, with the SVN group requiring the most analgesics and the EV group the least, highlighting the efficacy of the interventions.

Conclusions

Negative pressure irrigation (EndoVac) significantly reduced postoperative pain compared to conventional side-vented needle irrigation. These findings enhance understanding and guide evidence-based recommendations for optimizing endodontic procedures and prioritizing patient comfort and outcomes.

## Introduction

The prevalence of postoperative pain after root canal treatment varies from 3% to 58%, presenting a challenging issue [1,2]. Postoperative pain usually appears 24 hours after intervention, peaks within this time frame, and usually subsides within 48 hours, but it may persist intensely for several days in some cases [3-5]. The intensity of pain could vary from mild to severe and may necessitate medications for relief. The common cause of postoperative pain is local inflammatory changes arising from mechanical, chemical, and microbial insults to the periapex [6,7].

In endodontic practice, preoperative factors are critical for treatment planning and prognosis. Extensive research highlights the significance of preoperative pain, pulpal status, gender, tooth type, and periapical lesions in treatment success. Meanwhile, intraoperative factors such as microbial extrusion, dentinal debris, and mechanical injury must not be overlooked, as they can impact postoperative discomfort [8]. Therefore, addressing these iatrogenic factors is essential for minimizing discomfort and ensuring patient satisfaction throughout the endodontic procedure.

Mechanical debridement alone achieves approximately 60% cleanliness of the canal wall, leaving the remaining surfaces requiring additional disinfection. This underscores the importance of effective irrigation to address the intricate anatomical complexities of the root canal system [9,10]. Hence, the integration of irrigation and mechanical instrumentation works together to remove inflamed or necrotic tissue, microorganisms/biofilm, and debris from the root canal space [11]. Irrigation can be of positive, negative pressure, or activated irrigation techniques, which are conventional syringe-needle irrigation, EndoVac system, and EndoActivator, respectively. Irrigation activation devices, such as EndoActivator, are designed to improve the effectiveness of lateral canals with minimal extrusion compared to conventional methods [12].

Evidence suggests that machine-assisted agitation can indeed reduce postoperative discomfort [13]. While negative pressure irrigation has potential benefits in mitigating postoperative pain, it is often deemed clinically relevant in irrigant extrusion cases [14]. For instance, a study by Al-Zaka found that negative pressure irrigation led to decreased postoperative pain [15]. Nonetheless, it is important to acknowledge a limitation: the study participants did not report preoperative pain, potentially impacting the accuracy of postoperative pain observations.

Considering the potential efficacy of negative pressure irrigation in reducing postoperative pain, this clinical study aims to comparatively evaluate the effects of positive, negative pressure, and sonic-activated irrigation techniques on postoperative pain in teeth with symptomatic irreversible pulpitis and symptomatic apical periodontitis following two-visit endodontic therapy. The null hypothesis states that there will be no significant difference in positive pressure, negative pressure irrigation, or sonic-activated irrigation technique in causing postoperative pain.

## Materials and methods

**Figure 1:**
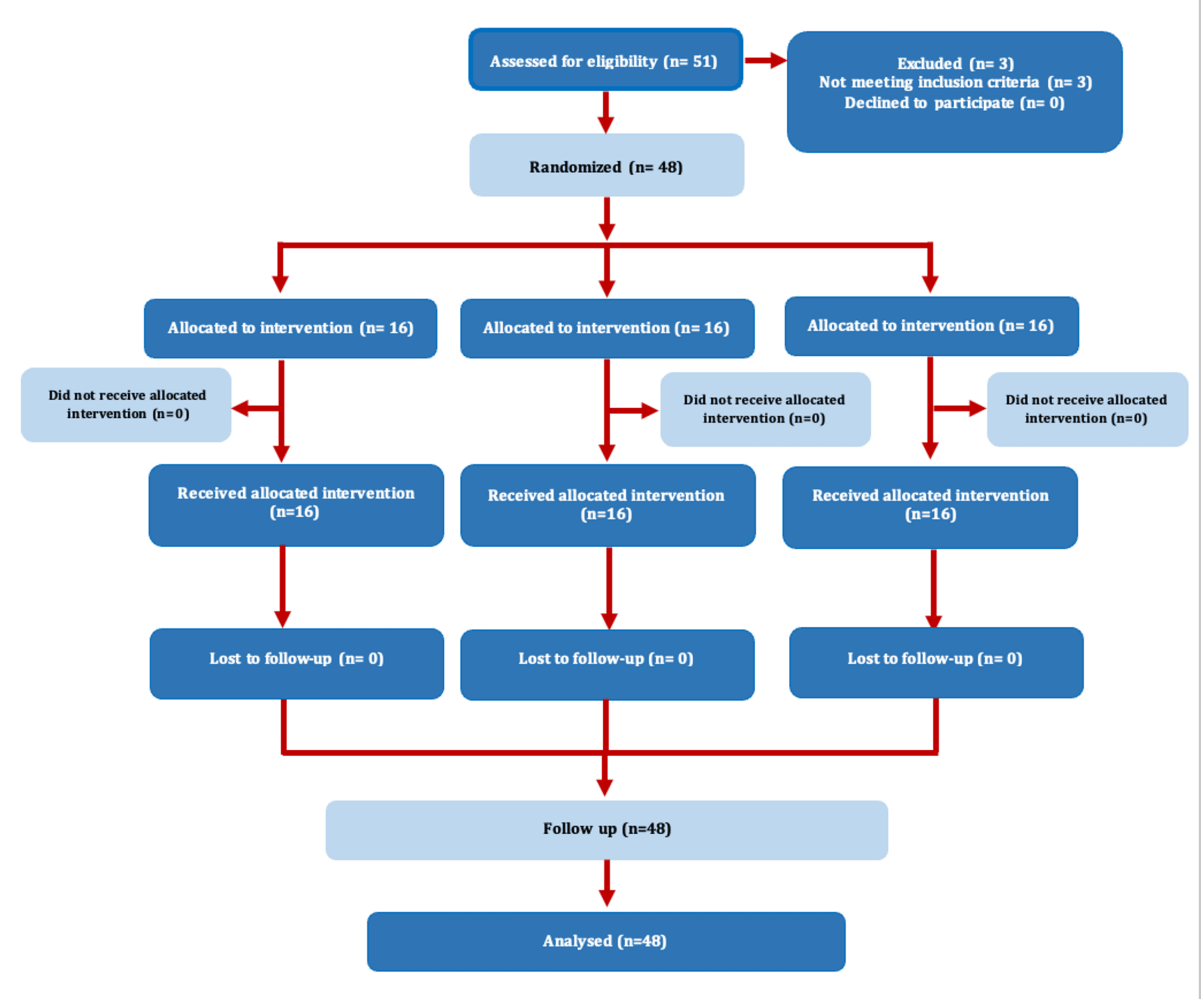
CONSORT flow diagram representing the flow of participants through each stage of a randomized trial CONSORT, Consolidated Standards of Reporting Trials

This double-blind, single-center, parallel-design randomized controlled trial was designed and reported according to the Consolidated Standards of Reporting Trials (CONSORT) 2010 guidelines (Figure 1).

The study, conducted at the Department of Dentistry, All India Institute of Medical Sciences, Jodhpur, India, received ethical approval from the Institutional Ethics Committee and was registered with the Clinical Trials Registry - India. The study was conducted in accordance with the Declaration of Helsinki and involved 48 volunteer patients meeting specific inclusion criteria from September 2022 to June 2023. To ensure comprehensive understanding, participants received verbal information and a bilingual written patient information sheet, addressing all queries satisfactorily.

Sample size calculation

The sample size, based on previous data by Ramamoorthi et al. [12], was calculated to be 14 teeth per group to achieve a statistical power of 80%, an effect size of 1.7, and a significance level of p < 0.05. This design, considering potential dropouts during follow-up, leads to a sample size of 16 teeth per group.

Patient selection and allocation

The study established specific inclusion and exclusion criteria to ensure a well-defined participant cohort. Inclusion criteria encompassed healthy individuals (ASA I) aged between 16 and 65 years, single-rooted teeth diagnosed with symptomatic irreversible pulpitis and symptomatic apical periodontitis, which was validated by the patient’s history, clinical evaluation with a positive electrical pulp test result (Vitality Scanner Model 2006, Sybron Endo, New York, United States), tenderness to percussion, and radiographic examination showing only slight periodontal ligament space widening without apical radiolucency. Patients exhibited a preoperative pain score ranging from moderate to severe (45-100 mm) on a Visual Analogue Scale (VAS). Conversely, exclusion criteria comprised individuals who had consumed analgesic or anti-inflammatory drugs within the last 12 hours that might alter their pain perception, pregnant or lactating individuals, teeth with calcified canals, preexisting periodontal diseases, root resorption, an immature or open apex, and previously treated or previously initiated therapy.

A total of 48 patients were assigned by the investigator into three experimental groups, with four groups of nine patients and two groups of six patients randomly allocated using the computer-generated block randomization method, with an allocation ratio of 1:1:1 within each block. The allocation concealment was ensured through the sequentially numbered, opaque, sealed envelopes (SNOSE) method, which effectively concealed the sequence until the interventions were assigned. A piece of paper containing a randomized group name was sealed in the dark envelope containing the respective serial number on it. The operator could not be blinded in the trial due to the nature of the interventions, but it was ensured that both the patient and assessor were blinded.

Prior to intervention, a comprehensive medical and dental history was gathered from each patient. Preoperative details, including age, gender, tooth number, and pain intensity measured on the VAS, were meticulously recorded in a pre-designed patient evaluation chart. Informed consent was obtained from willing participants after providing a detailed description of the treatment and study design.

Conducted in two visits by the same operator, all root canal therapy (RCT) procedures followed a systematic approach. The process included a pre-treatment rinse with 10 ml chlorhexidine gluconate mouthwash solution, administration of local anesthesia (2% lignocaine hydrochloride with 1:200,000 adrenaline), followed by the removal of caries. In cases of a missing proximal wall, restoration was carried out using a tooth-colored resin material (Spectrum Universal Microhybrid Composite Restorative), and isolation was achieved with a rubber dam (Hygienic, Coltene, and Whaledent Inc.).

Access to the pulp chamber was obtained, and pulp vitality confirmation involved observing bleeding from the canals. Working length was determined using an electronic apex locator (Propex Pixi, Dentsply Tulsa Dental, Tulsa, OK, USA) with stainless steel size #10 K-file, and subsequent canals were enlarged to three sizes larger than the first apical binding file, utilizing Hand ProTaper® Universal Files.

The irrigation procedures were implemented across three distinct groups using a 20-mL volume of a 3% NaOCl solution. In the Positive Pressure Irrigation (Conventional Side Vented Needle/SVN Group), the entire instrumentation process was complemented by irrigation with 3% NaOCl using a 30-G side vented needle, with residual solution flushed into canals 2 mm short of the working length. Moving to the Negative Pressure Irrigation (EndoVac/EV Group), the entire instrumentation process was supplemented with irrigation of the canal using 3% NaOCl through the master delivery tip, and a macrocannula was employed to flush out gross debris. The cleaning of the apical third of the root was conducted after chemomechanical preparation using a microcannula tip in a pecking motion until the remaining solution was completed. Each cycle of microcannula irrigation consisted of the tip being placed at full working length for six seconds and then withdrawn 2 mm from full working length for six seconds. This was repeated five times during a period of 30 seconds.

In the Sonic Activation Group (EndoActivator/EA), the entire instrumentation was supplemented by irrigation with 3% NaOCl using a 30-G side port needle. Following canal preparation, an EndoActivator was activated in the canal at a rate of 10,000 cycles per minute by inserting an EndoActivator tip (#25/.04), 2 mm short of the working length. This action involved 2-3 mm of vertical strokes agitating NaOCl for one minute, with NaOCl replenished after every minute until fully utilized.

The final irrigation included 5 mL of 17% ethylenediaminetetraacetic acid (EDTA), followed by 10 mL of distilled water, and the access cavity was sealed with a temporary filling material. A 400 mg of ibuprofen was provided to patients with explicit instructions for use solely in the event of severe pain. Subjects were instructed to complete a pain diary for evaluation of postoperative pain and frequency of analgesic intake at specific intervals (i.e., six, 24, 48, and 72 hours). Subjects were scheduled for a three-day post-treatment follow-up to facilitate pain diary retrieval and a comprehensive clinical evaluation to assess endodontic treatment completion.

Assessment of postoperative pain and analgesic intake

Inter-appointment pain and intake of analgesics were evaluated with a VAS at 6, 24, 48, and 72 hours after intervention. A horizontal ruler measuring 100 mm with marks spaced 10 mm apart served as the VAS. The pain levels will be documented as no pain (0-4 mm), mild pain (5-44 mm), moderate pain (45-74 mm), or severe pain (75-100 mm). All participants were trained to use the scale by an investigator. All information was tabulated by a single assessor.

Statistical analysis

All statistical analyses utilized IBM SPSS Statistics for Windows, Version 26.0 (Released 2019; IBM Corp., Armonk, NY, USA), and descriptive statistics were conducted for demographic variables, and normality was assessed using the Kolmogorov-Smirnov test. The intergroup analysis employed the Kruskal-Wallis test for non-normal data. Post hoc analyses were performed for both intra-group and inter-group comparisons when p-values were significant (p < 0.05).

## Results

Of the 51 individuals screened initially, 48 met the inclusion criteria and were enrolled in the study. Three potential participants were excluded due to ineligibility. Through random allocation, 48 patients were equally divided into three groups, each comprising 16 patients.

The study findings reveal diverse demographic characteristics and noteworthy insights into pre- and postoperative pain assessment across three distinct groups. The mean ages for Groups SVN, EV, and EA exhibited slight variations, ranging from 33.31 to 35.44 years, with an age range spanning 16 to 65 years. Gender distribution analysis demonstrated an equal representation of male and female patients in each group (Table 1).

**Table 1:** Demographic data of the intervention groups ^a^ Mean ± standard deviation Group SVN: conventional side-vented needle group; Group EV: negative pressure irrigation (EndoVac) group; Group EA: sonic activation (EndoActivator ) group

Demographic data	Group SVN	Group EV	Group EA
Age (years)	33.63 ± 14.44^a^	35.44 ± 12.30^a^	33.31 ± 15.49^a^
Gender			
Male	8	8	8
Female	8	8	8

In terms of preoperative pain, comparable baseline levels were observed among the groups. However, the postoperative pain assessment at different intervals showcased substantial variations. Notably, the mean postoperative pain scores were lowest in the EV group, followed by the EA group and the SVN group at all follow-up intervals. Intergroup analysis using the Kruskal-Wallis test identified statistically significant differences between the groups at six and 24 hours postoperatively (Table 2).

**Table 2:** VAS score, means, and standard deviation of the three groups Group SVN: conventional side-vented needle group; Group EV: negative pressure irrigation (EndoVac) group; Group EA: sonic activation (EndoActivator) group * Statistically significant VAS, Visual Analogue Scale

Time interval	Groups	Subjects	Mean	Standard deviation	p-value
Baseline (preoperative VAS score)	Group SVN	16	81.25	15.86	0.184
	Group EV	16	71.25	14.08	
	Group EA	16	75.63	14.59	
Postoperative six hours	Group SVN	16	36.25	20.61	0.018*
	Group EV	16	16.25	25.26	
	Group EA	16	26.88	22.43	
Postoperative 24 hours	Group SVN	16	18.75	16.68	0.018*
	Group EV	16	6.25	25.26	
	Group EA	16	11.88	13.77	
Postoperative 48 hours	Group SVN	16	6.25	10.82	0.281
	Group EV	16	1.88	5.44	
	Group EA	16	3.75	6.19	
Postoperative 72 hours	Group SVN	16	6.25	15.43	0.098
	Group EV	16	0	0	
	Group EA	16	1.25	3.42	

Post hoc tests elucidated a notable distinction in postoperative pain reduction between Group SVN and Group EV, emphasizing the impact of specific interventions. Further confirmation through the Bonferroni-corrected Mann-Whitney U test underscored a significant difference between Group SVN and Group EV at both six- and 24-hour postoperative intervals (Table 3).

**Table 3:** Statistical analysis evaluating intergroup analysis using the Bonferroni-corrected Mann-Whitney U test at six-hour and 24-hour intervals Group SVN: conventional side-vented needle group; Group EV: negative pressure irrigation (EndoVac) group; Group EA: sonic activation (EndoActivator) group * Statistically significant

Pairwise comparison	Postoperative six hours p-value	Postoperative 24 hours p-value
Group EV-Group EA	0.125	0.091
Group EV-Group SVN	0.005*	0.005*
Group EA-Group SVN	0.193	0.261

Analgesic requirements aligned with decreased postoperative pain, with nine, four, and two subjects in the SVN, EA, and EV groups, respectively, necessitating analgesics. There was a significant difference in the number of pills consumed between the SVN group and the EV group (Table 4).

**Table 4:** Distribution of analgesic requirements in all groups Group SVN: conventional side-vented needle group; Group EV: negative pressure irrigation (EndoVac) group; Group EA: sonic activation (EndoActivator) group * Statistically significant

Analgesics taken	Mean ± standard deviation	Median	Range	p-value
Group SVN	2.44 ± 2.68	2.5 (4)	0-9	0.012*
Group EV	0.19 ± 0.54	0 (0)	0-2	
Group EA	1.13 ± 1.58	0 (2)	0-4	

## Discussion

According to recent literature, there is currently no conclusive evidence demonstrating the superiority of negative pressure irrigation over syringe irrigation in the removal of hard tissue debris, soft tissue remnants, bacteria, or biofilm from the main root canal. One clinical trial and several ex vivo studies have found no significant difference between syringe irrigation and a range of other methods, including negative pressure irrigation, sonic, and ultrasonic activation, regarding the healing of apical periodontitis in teeth with a single root canal architecture [14]. Against this background, we have chosen to undertake a study to evaluate the effectiveness of positive pressure, negative pressure, and sonic-activated irrigation techniques in causing postoperative pain in teeth with symptomatic irreversible pulpitis and symptomatic apical periodontitis following a two-visit RCT. The objective of this study is to provide empirical data to assist practitioners in choosing irrigation techniques, ultimately leading to improved patient outcomes with decreased post-treatment discomfort.

Our study specifically included symptomatic irreversible pulpitis and symptomatic apical periodontitis cases, which were associated with moderate to severe preoperative pain on the VAS. We carefully selected these criteria based on their proven ability to predict postoperative pain, as demonstrated in previous research [16].

We acknowledged the significant correlation between preoperative and postoperative pain and implemented intentional measures to identify any variables that could mask the effects of irrigation techniques on postoperative pain. Moreover, we excluded participants who had taken pain medications within 12 hours before treatment to ensure a precise diagnosis and avoid any potential impact of drugs, which has been emphasized in earlier studies [8].

The decision to utilize a parallel group design in this study was made with great care, as it was acknowledged that discomfort in one tooth may impact the perception of pain in another. Consequently, participants with two affected teeth were intentionally excluded from the analysis. In the majority of clinical studies with a comparable focus on intraoperative pain evaluation, it is customary to use single-rooted teeth [17,18]. The rationale behind this conscious decision is to minimize the likelihood of iatrogenic complications that may arise from intricate root canal architecture or potential canal omissions.

To accurately record pain levels, we utilized the VAS, which is a widely accepted and established method for assessing pain levels during or following root canal treatment, as it is reliable, methodologically sound, and straightforward to administer in pain evaluation [19,20].

Although typically not severe, patients may experience postoperative discomfort after undergoing root canal treatment. According to a systematic review, the incidence of pain is highest within the first 24 hours post-treatment and decreases to 10% or less after a week. It is worth noting that different instruments and techniques used during root canal preparation can result in varying levels of debris extrusion, which may contribute to differences in postoperative pain [21].

To standardize the process of root canal preparation, we utilized the ProTaper universal file system, which is renowned for its effectiveness, versatility, and shaping ability [22]. Our primary choice of irrigant was sodium hypochlorite (NaOCl) due to its superior ability to dissolve organic tissues. Given the limitation of an incomplete smear layer elimination, EDTA was introduced as a supplemental solution.

Obturation and post-endodontic restoration were completed during the second sitting of our study. The purpose of this careful approach was to minimize the possibility of postoperative discomfort that could arise from the extrusion of filling materials [12].

**Figure 2:**
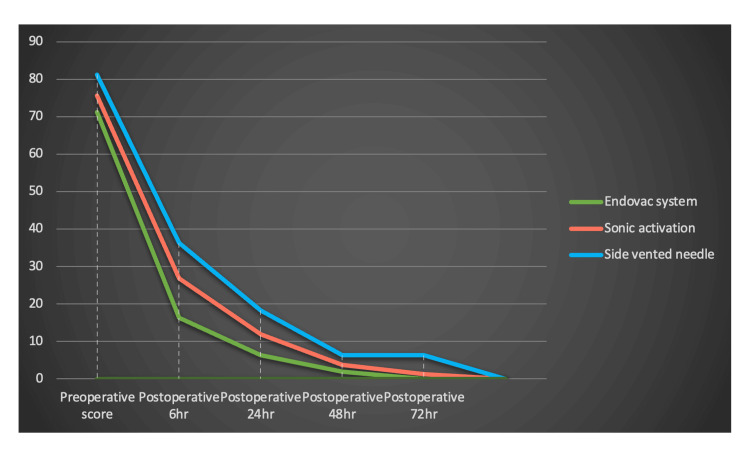
Graphical representation of reduction in pain scores

The clinical trial recruited a total of 48 participants, who were evenly distributed among three groups. Moreover, the trial aimed to maintain a well-balanced age distribution, and the gender distribution was also equally represented, with a 1:1 male-to-female ratio across all groups. Prior to the interventions, baseline pain levels were assessed using the VAS and found to be similar among Groups SVN, EV, and EA, with scores of 81.25, 71.25, and 75.63, respectively. Following the procedures, all groups experienced a consistent reduction in pain over different time intervals, as depicted in Figure 2.

During the zero- to six-hour interval, 72.9% of patients reported pain, with 31.2% belonging to the SVN group, 12.5% to the EV group, and 29.1% to the EA group. In the six- to 24-hour interval, out of 52% of patients, 25%, 6%, and 20.8% were associated with the SVN, EV, and EA groups, respectively. For the 24- to 48-hour interval, among 27% of patients, 10.4%, 4%, and 10.4% were linked to the SVN, EV, and EA groups. Lastly, in the 48- to 72-hour interval, among 12.5% of patients, 8.3%, 0%, and 4.1% were attributed to the SVN, EV, and EA groups.

The findings distinctly indicate that the EndoVac group exhibited the most significant pain reduction, starting at the six-hour interval (12.5%) and consistently reaching 0% at the 72-hour interval. This pattern underscores the EV group’s remarkable efficacy in minimizing pain, with notable reductions at the six- and 24-hour intervals compared to the SVN group.

The mechanical EndoActivator system consists of a handpiece and variously sized polymer tips designed to produce a hydrodynamic phenomenon and activate intracanal reagents safely. The flexible polymer tips are driven by an ergonomic, cordless, contra-angled sonic handpiece set at 2,000, 6,000, and 10,000 CPM, significantly enhancing three-dimensional disinfection. The EndoActivator effectively dislodges biofilms from complex root canal systems, removes the smear layer, and debrides uninstrumentable areas. The device generates intracanal waves and fluid activation, producing bubbles that enlarge, compress, and implode, creating up to 30,000 shockwaves per implosion. These powerful shockwaves penetrate biofilms and clean surfaces, a phenomenon often observed as a debris cloud in fluid-filled pulp chambers [23].

The EndoVac™ apical negative pressure irrigation system comprises three integral components: the macrocannula, microcannula, and Master Delivery Tip. The Master Delivery Tip accommodates an irrigant syringe, delivering its content through a 20-gauge needle. The macrocannula serves the dual purpose of irrigating the pulp chamber and directing the irrigant toward the axial wall, facilitating the suction of material up to the middle third segments of the canal. The microcannula, equipped with 12 small pores, consistently dispenses waste throughout its operation. The 0.32 mm zero-taper stainless-steel microcannula features four sets of three laser-cut offset holes, each measuring 100 μm in diameter and spaced 100 μm apart near the closed end. The microcannula’s internal lumen, with an ISO size of 0.20 mm internal diameter, prevents clogging through the filtering action of these pores. The microcannula is used to irrigate the apical third of the canal when positioned at its working length [24].

During our study, all irrigation methods were utilized according to the manufacturer’s instructions. The SVN group implemented a safety measure to maintain a 2 mm distance between the needle tip and the apex, which is consistent with similar research that utilizes a range of 1.5-3 mm ahead of the working length [25]. In line with the manufacturer’s recommendations, the EndoActivator tip was intentionally held back by 2 mm from the working length, while the EndoVac microcannula tip was situated at the full working length [26].

The amount of extruded irrigant has been shown to be a crucial factor in postoperative pain, as it can initiate chemical irritation of periradicular tissues, which leads to discomfort and pain for the patient, as noted by Seltzer and Naidorf in their study [27]. Therefore, it is important to carefully consider the placement of irrigation needles used during endodontic procedures to minimize the risk of complications and ensure optimal patient outcomes.

The reason behind the noticeable reduction in postoperative pain associated with the EndoVac system can be attributed to its unique mechanism. By utilizing negative pressure through a microcannula, the system allows for precise delivery of irrigant to the apical third while also minimizing extrusion. Additionally, its ability to prevent air entrapment distinguishes it from traditional irrigation methods. This finding is supported by previous studies that emphasize the effectiveness of the EndoVac system in maintaining irrigation within the root canal, ultimately leading to a decrease in postoperative discomfort [17,28-30].

Romualdo et al.’s recent systematic review underscores the efficacy of apical negative pressure irrigation in preventing apical extrusion compared to positive pressure methods [31]. Interestingly, a study included in the review found no significant distinction between conventional irrigation and apical negative pressure [32].

Yost et al. studied the effect of various methods on NaOCl apical extrusion and concluded that EndoVac produced significantly less extrusion as compared to side-vented irrigation. However, no significant difference was observed between the EndoVac and EndoActivator groups [33].

Numerous studies have consistently shown that the use of the EndoVac system results in enhanced irrigant penetration up to the apical third of the root canal, accompanied by significantly improved debridement of the apical third. These findings have been reported in studies conducted by Nielsen and Craig Baumgartner [34], Tay et al. [35], and Munoz and Camacho-Cuadra [36]. The results of these studies highlight the potential of the EndoVac system as an effective tool for improving endodontic procedures and outcomes.

Different pain relievers have been used to ease discomfort after RCT, and NSAIDs are typically the preferred option. Ibuprofen is a noteworthy medication within this category, commonly used for managing postoperative pain and frequently studied in relation to its effectiveness [37]. In our research, we selected ibuprofen as the recommended medication following treatment.

**Figure 3:**
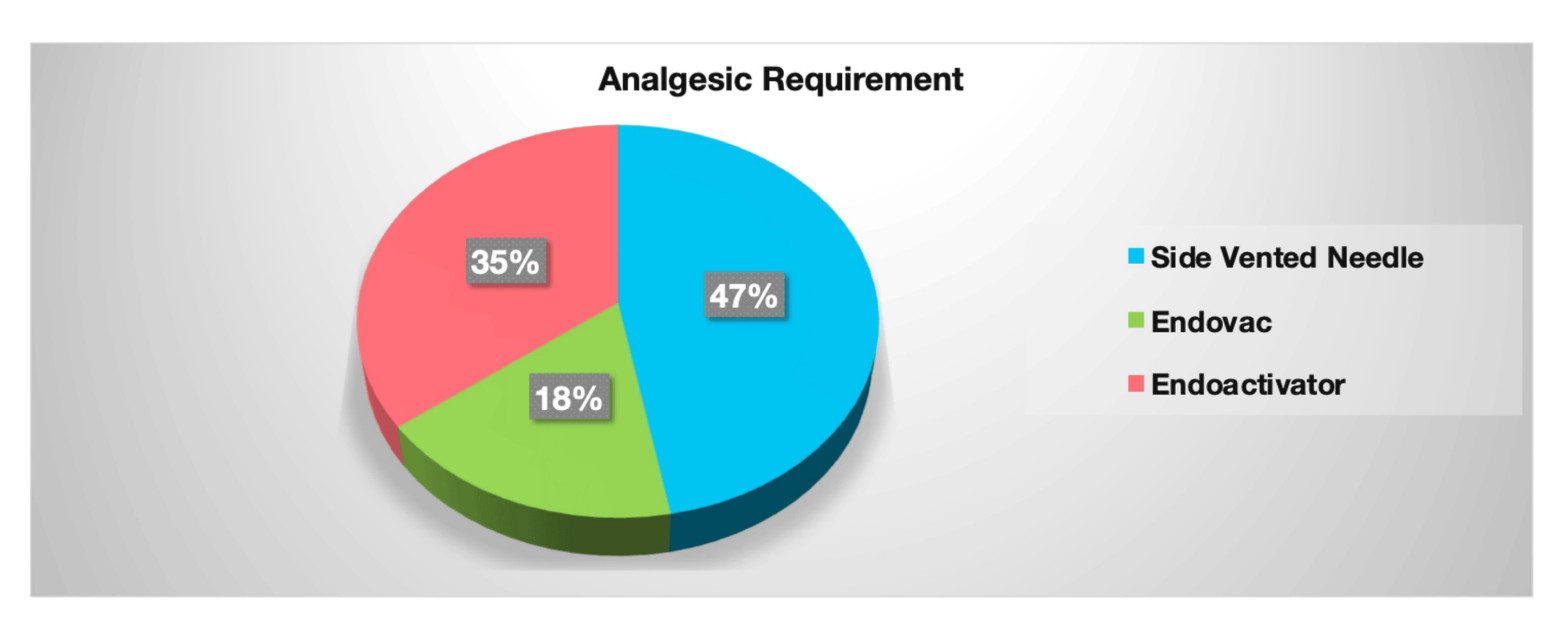
Pie chart representing the requirements of analgesics

Our investigation revealed that 37.5% of patients felt the need to use analgesic medication after the procedure. Of this group, 47% of patients in the SVN group, 35% in the EA group, and 18% in the EV group required analgesics (Figure 3).

However, it is important to note that relying solely on the quantity of analgesics taken to measure pain levels may introduce bias. For instance, some patients may experience severe pain but choose not to take medicine, while others may take medication for milder pain. Therefore, our study examined the relationship between analgesic consumption and pain intensity to provide a more accurate representation of the pain experienced by patients. The findings show a significant association between pain severity and the number of pills taken in the EndoVac and SVN groups. It is clear from the results that the demand for analgesics was lower in the EndoVac group, emphasizing the importance of incorporating advanced irrigation techniques like EndoVac to ensure elevated patient comfort and optimal outcomes in RCT.

Upon conducting a comprehensive analysis of the study, it was determined that the EndoVac negative pressure irrigation system surpasses both Sonic Activation and Side Vented Needle irrigation protocols. EndoVac’s ability to significantly decrease postoperative discomfort resulted in a decreased need for analgesic medications, ultimately demonstrating its clinical effectiveness. These findings emphasize the significance of incorporating advanced irrigation techniques, such as EndoVac, to not only guarantee elevated patient comfort but also achieve optimal outcomes in RCT.

The strength of the current study lies in its design. In the study design and reporting stages, CONSORT statements were followed [38]. It is important to acknowledge the limitations of the present study. Pain is a subjective experience, and there are many known and unknown factors that can affect it, which are difficult to control in a clinical setting. The operator giving the intervention was not blinded to the groups because of the clinical nature of the trial, which could be a limitation. The clinicians’ awareness of the intervention applied might introduce bias, although this was mitigated by having the outcome assessor blinded to the intervention allocation. Another constraint is the limited sample size, emphasizing the need for future research with larger cohorts to thoroughly evaluate the incidence and intensity of postoperative pain using positive pressure, negative pressure, and sonic-activated irrigation for more robust results.

## Conclusions

Based on the findings of the current study, negative pressure irrigation (EndoVac) significantly reduced postoperative pain and analgesic requirements compared to conventional side-vented needle irrigation. It is safe to employ a negative apical pressure irrigation system for antimicrobial debridement throughout the full working length.

These findings enhance understanding and guide evidence-based recommendations for optimizing endodontic procedures and prioritizing patient comfort and outcomes.
